# Epidemiology, Microbiology and Mortality Associated with Community-Acquired Bacteremia in Northeast Thailand: A Multicenter Surveillance Study

**DOI:** 10.1371/journal.pone.0054714

**Published:** 2013-01-18

**Authors:** Manas Kanoksil, Anchalee Jatapai, Sharon J. Peacock, Direk Limmathurotsakul

**Affiliations:** 1 Department of Pediatrics, Sappasithiprasong Hospital, Ubon Ratchathani, Thailand; 2 Mahidol-Oxford Tropical Medicine Research Unit, Mahidol University, Bangkok, Thailand; 3 Department of Microbiology and Immunology, Mahidol University, Bangkok, Thailand; 4 Department of Tropical Hygiene, Faculty of Tropical Medicine, Mahidol University, Bangkok, Thailand; 5 Department of Medicine, Cambridge University, Addenbrooke’s Hospital, Cambridge, United Kingdom; Wake Forest University School of Medicine, United States of America

## Abstract

**Background:**

National statistics in developing countries are likely to underestimate deaths due to bacterial infections. Here, we calculated mortality associated with community-acquired bacteremia (CAB) in a developing country using routinely available databases.

**Methods/Principal Findings:**

Information was obtained from the microbiology and hospital database of 10 provincial hospitals in northeast Thailand, and compared with the national death registry from the Ministry of Interior, Thailand for the period between 2004 and 2010. CAB was defined in patients who had pathogenic organisms isolated from blood taken within 2 days of hospital admission without a prior inpatient episode in the preceding 30 days. A total of 15,251 CAB patients identified, of which 5,722 (37.5%) died within 30 days of admission. The incidence rate of CAB between 2004 and 2010 increased from 16.7 to 38.1 per 100,000 people per year, and the mortality rate associated with CAB increased from 6.9 to 13.7 per 100,000 people per year. In 2010, the mortality rate associated with CAB was lower than that from respiratory tract infection, but higher than HIV disease or tuberculosis. The most common causes of CAB were *Escherichia coli* (23.1%), *Burkholderia pseudomallei* (19.3%), and *Staphylococcus aureus* (8.2%). There was an increase in the proportion of Extended-Spectrum Beta-Lactamases (ESBL) producing *E. coli* and *Klebsiella pneumoniae* over time.

**Conclusions:**

This study has demonstrated that national statistics on causes of death in developing countries could be improved by integrating information from readily available databases. CAB is neglected as an important cause of death, and specific prevention and intervention is urgently required to reduce its incidence and mortality.

## Introduction

Community-acquired bacteremia (CAB) is a leading cause of morbidity and mortality in the general population. In high-income countries, the rate of hospitalization due to CAB is around 77 to 92 per 100,000 people per year, and the associated overall 30-day mortality is around 13 to 19% [1], [2], [3], [4], [5]. There has been a documented increase in the incidence rate of CAB over the last 15 years [2], and bacteremia is now the eleventh most frequent cause of death in the United States [6]. There are very few population-based studies evaluating incidence and trends of CAB in low and middle-income countries, an important omission since such information is required for strategic planning of available healthcare resources, together with disease prevention programs.

In many developing countries, there are a growing number of diagnostic microbiological laboratories that provide routine culture services [7], [8]. A blood culture positive for pathogenic bacteria that has been collected within the first 2 days of admission from a patient without a history of previous hospitalization indicates CAB [1], [2], [3], [4], [5]. Information collected by laboratory databases could be used to estimate the incidence of serious bacterial infections in the general population, although this is rarely used in developing countries as a source of data for local, regional or national statistics, nor used to inform public health policy. The mortality rate associated with bacteremia may also be discrepant between that derived from hospital data and figures from national statistics based on death certification. For example, in Thailand, CAB is not an important cause of death in national statistics [9], but this is inconsistent with a number of studies showing an increasing incidence of bacterial infections in NE Thailand [10], [11], [12]. We hypothesized that mortality due to CAB in Thailand has been underestimated, and that routinely collected data from the microbiology laboratory can be used to inform public health policy. In this study, our objectives were to estimate the incidence, trends and mortality associated with CAB in northeast Thailand using multiple sources of routine surveillance data including routine microbiology and hospital admission databases from provincial hospitals, and to compare these with national death registry from the Ministry of Interior, Thailand.

## Materials and Methods

### Study Population

Northeast Thailand consists of 20 provinces, covers 170,226 km^2^ and had an estimated population in 2010 of 21.4 million [13]. Each province has a provincial hospital that provides care to people living within its catchment area and acts as a referral hospital to one or more smaller district hospitals. Severely ill patients presenting to district hospitals are often referred to provincial hospitals. Provincial hospitals are equipped with a microbiology laboratory that provides a bacterial culture service, while district hospitals do not have microbiology facilities. All microbiology laboratories in provincial hospitals follow the standard methodologies for bacterial identification and susceptibility testing provided by the Bureau of Laboratory Quality and Standards, Ministry of Public Health, Thailand. The majority of the population in northeast Thailand live in rural settings and most adults (around 80%) are engaged in agriculture, particularly rice farming.

### Study Design

We conducted a retrospective, multicenter surveillance study of all provincial hospitals in northeast Thailand. The director of each hospital was contacted and given information on the study. For those hospitals that agreed to participate, data were collected from the microbiology and hospital databases between Jan 2004 to Dec 2010. Admission number (AN) was used as a record linkage between the two databases, and hospital number (HN) was used to identify individuals who had repeated admissions. The death registry for northeast Thailand between Jan 2004 to Jan 2011 was obtained from the Ministry of Interior, Thailand, and used to identify patients who were discharged from hospital and died within 30 days of admission date. Ethical permission for this study was obtained from the Ethical and Scientific Review Committees of the Faculty of Tropical Medicine, Mahidol University, and of the Ministry of Public Health, Thailand. Written consent was given by the director of the hospitals to use the routine hospital database for research. Consent was not sought from the patients as this was a retrospective study, and the Ethical and Scientific Review Committees approved the process.

### Data Collection

The microbiology laboratory data collected was HN, AN, specimen type, specimen date, culture result and antibiotic susceptibility profile (antibiogram). Hospital data were collected from the routine in-patient discharge report (Report 501), which is regularly completed by attending physicians and reported to Ministry of Public Health, Thailand, as national morbidity and mortality reporting system [14]. The data collected was HN, AN, national identification 13-digit number, gender, age, admission date, discharge date, diagnoses according to ICD-10 (*International Classification of Diseases,* 10^th^ revision) codes, and outcome. A single outcome variable is required by this reporting system, which is completed by the attending physicians and categorized as cured, improved, not improved, transfer to another hospital, refusal of treatment, or died [14]. Date of death was also extracted from this record. Data collected from the death registry obtained from the Ministry of Interior was national identification included 13-digit number, cause of death according to the ICD-10 code, and date of death. Population data by age and sex for each province in Thailand for the years 2004–2010 were obtained from the Department of Provincial Administration, Thailand [13].

### Definitions

Bacteremia was classified as CAB, healthcare-associated bacteremia (HCAB) or hospital-acquired bacteremia (HAB) [1], [2], [15]. CAB was defined in patients who had pathogenic organisms isolated from blood taken in the first 2 days of admission and without a hospital stay within 30 days prior to the admission. HCAB was defined in patients who had pathogenic organisms isolated from blood taken in the first 2 days of admission and with a hospital stay within 30 days prior to the admission. HAB was defined in patients who had pathogenic organisms isolated from blood cultures taken 2 or more days after admission. Because of the difficulty in establishing their clinical significance, organisms frequently associated with contamination including coagulase-negative staphylococci, viridans group streptococci, *Corynebacterium* spp., *Bacillus* spp., *Diptheroid* spp., *Micrococcus* spp. and *Propionibacterium* spp. were excluded from the analysis. All patients with bacteremia caused by *B. pseudomallei* were categorized as CAB because this organism is not a cause of HAB or HCAB [16]. Polymicrobial infection was defined in CAB patients who had more than one species of pathogenic organisms isolated from blood cultures taken within the first 2 days of admission. Patients with a first episode of CAB were evaluated in relation to epidemiology, microbiology and mortality.

30-day mortality was determined on the basis of a record of death within 30 days of admission in the routine hospital database or by a record of death within that period in the national death registry. The incidence rate of CAB was calculated as the number of CAB identified in the participating hospitals per 100,000 people per year. Mortality rate attributable to CAB was calculated as the number of CAB patients who died within 30 days of the admission per 100,000 people per year.

Mortality rate were calculated for six other important infectious diseases or disease syndromes [17], as follows: HIV disease, tuberculosis, lower respiratory tract infection, diarrhea and malaria [6]. Death due to these diseases was defined in patients who were admitted to the study hospitals, died within 30 days of admission, and had the underlying cause of death based on ICD-10 codes of HIV disease (B20–24), tuberculosis (A15–19), lower respiratory tract infection (J09–18), diarrhea (A09), malaria (B50–54), or measles (B05) [6], after excluding those who died within 30 days due to CAB as described above.

### Statistical Analysis

Chi-square test for trend was used to assess change in proportion over time. Poisson regression models were used to calculate rate ratios. CIs were calculated using the Poisson method. The Fisher’s exact test was used to compare categorical variables. All analyses were performed using the STATA version 12.0 (StataCorp LP, College station, Texas).

## Results

The 20 provincial hospitals in northeast Thailand were contacted to participate in this study. Agreement was obtained from 15 (75%) hospitals, of which 10 had microbiological laboratory and hospital databases as electronic files in a readily accessible format. Of the 10 hospitals included in the analysis, 3 (30%) had data available for the period 2004–2010, 1 (10%) between 2006–2010, 2 (20%) between 2007–2010, 3 (30%) between 2008–2010, and 1 (10%) between 2009–2010 (Table 1). A total of 1,969,652 admission records were evaluated, of which 21,438 (1.1%) had at least one blood culture positive for pathogenic organisms during admission. A total of 3,451 (16.1%) cases were defined as hospital-acquired bacteremia (HAB), 2,302 (10.7%) cases were healthcare-associated bacteremia (HCAB), and 15,685 (73.2%) cases were community-acquired bacteremia (CAB). Multiple episodes of CAB were noted in 390 patients, and only the first episode of CAB in 15,251 patients was included in further analyses.

**Table 1:** Incidence of community-acquired bacteremia (CAB) and associated death rate between 2004–2010 in northeast Thailand.

Year	Total number of hospitals with available data *	Total number of population at risk (per year)	Total number of hospital admission	Hospital admission rate (per 100,000 people per year)	Total numberof patientswith CAB	Deaths associated with CAB	30-day mortality associatedwith CAB	Incidence rate for CAB (per 100,000 people per year)	Mortality rate associated with CAB (per 100,000 people per year)
2004	3	4,271,105	129,376	3029.1	712	293	41.2%	16.7	6.9
2005	3	4,286,718	138,816	3238.3	964	370	38.4%	22.5	8.6
2006	4	4,900,484	187,812	3832.5	1,147	430	37.5%	23.4	8.8
2007	6	5,879,985	241,208	4102.2	1,935	731	37.8%	32.9	12.4
2008	9	8,831,777	372,564	4218.4	2,907	1154	39.7%	32.9	13.1
2009	10	10,395,646	453,791	4365.2	3,598	1310	36.4%	34.6	12.6
2010	10	10,442,963	445,907	4269.9	3,988	1434	36.0%	38.2	13.7
Overall	10	49,008,680	1,969,474	4018.6	15,251	5,722	37.5%	31.1	11.7

* Data were from Chaiyaphum, Sisaket and Ubon Ratchathani hospitals from 2004 to 2010, Loei hospital from 2006 to 2010, Mahasarakham and Nakhon Phanom hospitals from 2007 to 2010, Nong Khai, Udon Thani and Yasothon hospitals from 2008 to 2010, and Buriram hospital from 2009 to 2010.

### Incidence of CAB

The average incidence rate for CAB in northeast Thailand during the 7-year study period was 31.1 cases per 100,000 people per year, although there was a marked increase in rate over time (Table 1). The incidence rate of CAB increased from 16.7 to 38.2 per 100,000 people per year between 2004 and 2010 (p<0.001). This rise over time was observed in every province except Buriram and Yasothorn, where data were only available for the last 2 and 3 years, respectively (data not shown). During the same period, the provincial hospital admission rate rose from 3,029.1 in 2004 to 4,269.9 per 100,000 people in 2010 (p<0.001), and an incidence of CAB per 1,000 admissions rose from 5.5 in 2004 to 8.9 in 2010 (p<0.001).

### Demographic Risk Factors for CAB

**Figure 1:**
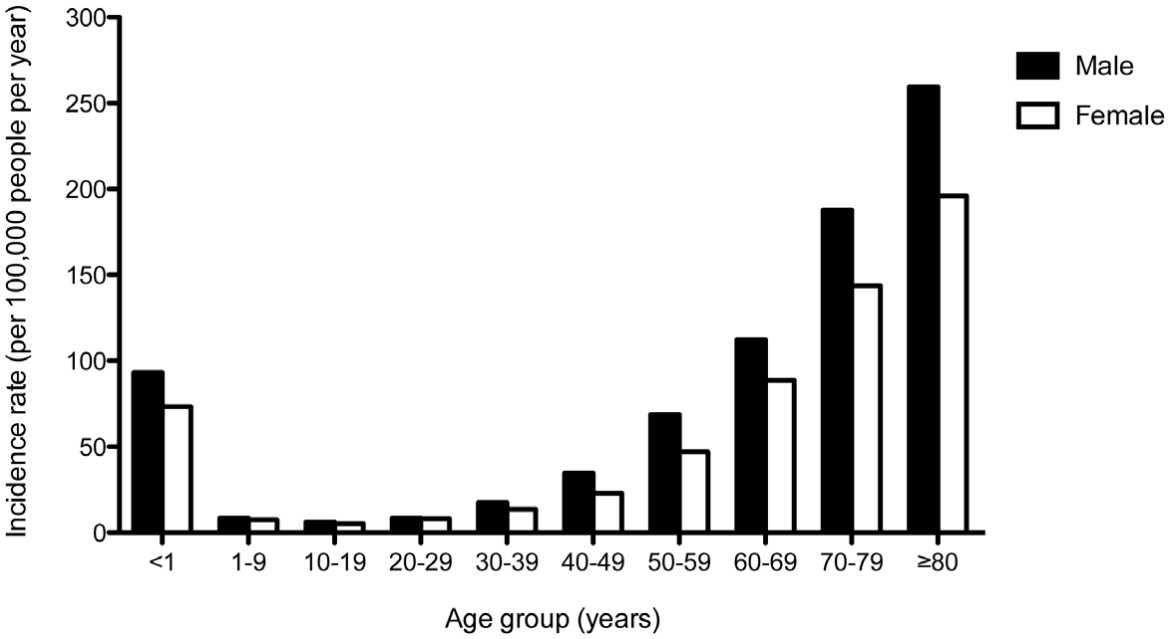
Age- and gender- specific incidence rate of community-acquired bacteremia (CAB), northeast Thailand, 2004–2010. CAB was defined in patients who had pathogenic organisms isolated from blood taken in the first 2 days of admission and without a hospital stay within 30 days prior to the admission. The incidence rate of CAB was calculated as the number of CAB identified in the participating hospitals per 100,000 people per year.

**Figure 2:**
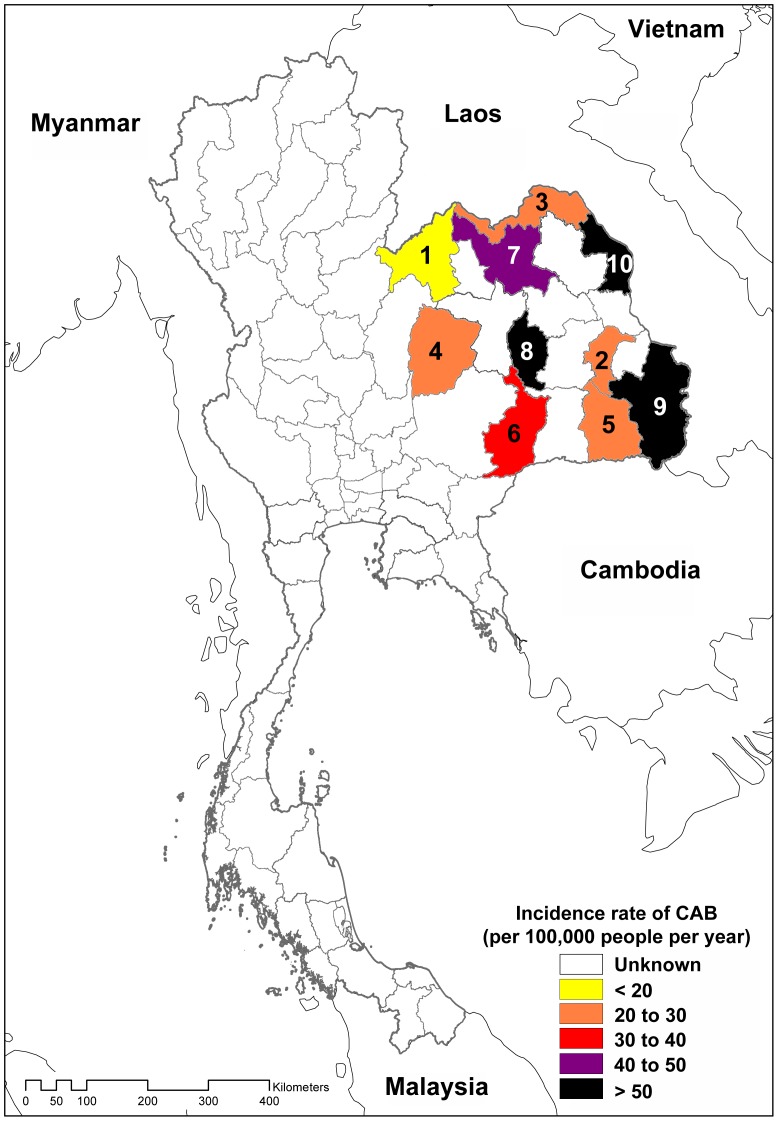
Map of estimated incidence rates for community-acquired bacteremia (CAB), northeast Thailand, 2010. Provinces are ordered by estimated incidence rates of CAB. Provincial codes: 1. Loei, 2. Yasothon, 3. Nong Khai, 4. Chaiyaphum, 5. Sisaket, 6. Buriram, 7. Udon Thani, 8. Mahasarakham, 9. Ubon Ratchathani, and 10. Nakhon Phanom.

Of 15,251 patients with a primary episode of CAB, 8,329 (54.6%) were male and 6,922 (45.4%) were female. Median age was 57 years (range, 0–104 years; interquartile range [IQR], 41–70 years), and 1,231 (8.1%) were younger than 15 years of age. The overall incidence rate of CAB was higher in males than in females (34.0 vs. 28.2 per 100,000 people, rate ratio 1.20; 95% CI 1.17 to 1.24, p<0.001), and this was observed in most age groups (Figure 1). The incidence rate of CAB was highest in infants and the elderly. The incidence rate of CAB was 83.5 in children younger than 1 year of age, falling to less than 10 per 100,000 people between the age of 1 and 30 years. The annual incidence of CAB rose from 15.5 in people between the ages of 30–39 years to 221.8 per 100,000 for people who were older than 80 years old. The incidence of CAB also varied between provinces. In 2010, the lowest incidence was observed in Loei province at 18.4 per 100,000 people, while the highest incidence was observed in Nakhon Phanom province at 57.8 per 100,000 people (Figure 2).

### Pathogenic Organisms Associated with CAB

Of all pathogenic organisms isolated, 10,844 (71.1%) were Gram-negative bacteria, 3,692 (24.2%) were Gram-positive bacteria, 175 (1.1%) were fungi, 25 (0.2%) were *Mycobacterium* spp., and 515 (3.4%) were polymicrobial (Table 2). The most common pathogens identified were *Escherichia coli* (23.1%), *Burkholderia pseudomallei* (19.3%), and *Staphylococcus aureus* (8.2%). Gram-positive bacteria were more common in children younger than 15 years old than in adults (41.2% vs. 22.7%, p<0.001). The common pathogens in infants younger than 1 year old were *S. aureus* (15.1%), *E. coli* (10.9%), and *Acinetobacter* spp. (8.9%), and those in children age from 1 to 15 years old were *S. aureus* (24.0%), *B. pseudomallei* (14.3%) and *Pseudomonas* spp. (8.9%). *Salmonella* enterica serovar Typhi was isolated from only 7 patients during the study period. Extended-Spectrum Beta-Lactamases (ESBL) producing strains were found in 11.8% (415/3,525) of *E. coli* and 11.4% (118/1,032) of *Klebsiella pneumoniae*, while methicillin resistance was detected in 5.7% (71/1,247) of *S. aureus*.

**Table 2:** Pathogenic organisms isolated from 15,251 patients with primary episode of community-acquired bacteremia (CAB) in northeast Thailand between 2004 and 2010.

Code	Organisms	Infants (age <1 yr)	Children (age 1–15 yr)	Adults (age >15 yr)
0000a	Gram negative bacteria	252 (50.1%)	395 (54.3%)	10,197 (72.7%)
0000b	* Salmonella enterica*			
0001	* S. enterica* serotype Typhi	–	4 (0.5%)	3 (0.02%)
0002	Non-typhoidal *Salmonella*	15 (3.0%)	43 (5.9%)	481 (3.4%)
0002a	Non-salmonella enterobacteiaceae			
0002b	* Escherichia coli*			
0003	ESBL –ve	51 (10.1%)	34 (4.7%)	3,025 (21.6%)
0004	ESBL +ve	4 (0.8%)	4 (0.5%)	407 (2.9%)
0004a	* Klebsiella pneumonia*			
0005	ESBL –ve	22 (4.4%)	13 (1.8%)	879 (6.3%)
0006	ESBL +ve	9 (1.8%)	7 (1.0%)	102 (0.7%)
0007	* Klebsiella* spp	6 (1.2%)	2 (0.3%)	256 (1.8%)
0008	* Enterobacter* spp	23 (4.6%)	18 (2.5%)	223 (1.6%)
0009	* Proteus* spp	3 (0.6%)	–	129 (0.9%)
0010	* Shigella* spp	–	1 (0.1%)	3 (0.02%)
0020	Other Enterobacteriaceae	4 (0.8%)	3 (0.4%)	109 (0.8%)
0020a	Other Gram-negative			
0021	* Burkholderia pseudomallei*	8 (1.6%)	104 (14.3%)	2,831 (20.2%)
0022	* Pseudomonas* spp	37 (7.4%)	65 (8.9%)	716 (5.1%)
0023	* Acinetobacter* spp	45 (8.9%)	54 (7.4%)	409 (2.9%)
0024	* Achromobacter* spp	1 (0.2%)	2 (0.3%)	194 (1.4%)
0025	* Aeromonas* spp	1 (0.2%)	5 (0.7%)	142 (1.0%)
0026	* Haemophilus* spp	14 (2.8%)	14 (1.9%)	29 (0.2%)
	* Vibrio* spp	1 (0.2%)	3 (0.4%)	15 (0.1%)
0027	* Moraxella* spp	1 (0.2%)	1 (0.1%)	27 (0.2%)
0028	* Neisseria* spp	–	–	5 (0.04%)
0029	Other Gram negatives	6 (1.2%)	7 (1.0%)	179 (1.3%)
0030	Unspecified Gram negatives	1 (0.2%)	11 (1.5%)	33 (0.2%)
0031	Gram positive	212 (42.1%)	303 (41.6%)	3,177 (22.7%)
0050a	* Staphylococcus aureus*			
0050b	Methicillin-susceptible	71 (14.1%)	172 (23.6%)	933 (6.7%)
0051	Methicillin-resistant	5 (1.0%)	3 (0.4%)	63 (0.4%)
0052	* Streptococcus pneumoniae*	37 (7.4%)	46 (6.3%)	345 (2.5%)
0053	Group A streptococcus	11 (2.2%)	16 (2.2%)	418 (3.0%)
0054	Group B streptococcus	23 (4.6%)	7 (1.0%)	339 (2.4%)
0055	Group D streptococcus (non-Enterococci)	13 (2.6%)	9 (1.2%)	271 (1.9%)
0056	Other streptococci	9 (1.8%)	19 (2.6%)	338 (2.4%)
0057	* Enterococcus* spp	35 (7.0%)	10 (1.4%)	279 (2.0%)
0058	Other Gram positives	1 (0.2%)	1 (0.1%)	6 (0.04%)
0059	Unspecified Gram positives	7 (1.4%)	20 (2.7%)	185 (1.3%)
0060	Fungi	2 (0.4%)	8 (1.1%)	165 (1.2%)
0060a	Cryptococcus spp	–	3 (0.4%)	135 (1.0%)
0061	Candida spp	2 (0.4%)	4 (0.5%)	19 (0.1%)
0062	Penicillium spp	–	1 (0.1%)	11 (0.1%)
0063	*Mycobacterium* spp.	–	–	25 (0.2%)
0071	Polymicrobial infections	37 (7.4%)	22 (3.0%)	456 (3.3%)
0099	Overall	503 (100%)	728 (100%)	14,020 (100%)

There was no difference in the pattern of common pathogens identified between different provinces or over the study period, but there was an increase in the proportion of ESBL producing *E. coli* and *K. pneumoniae* over time. The proportion of ESBL producing *E. coli* rose from 2.9% (5/171) in 2004 to 18.0% (156/868) in 2010 (p<0.001). The proportion of ESBL producing *K. pneumoniae* was 10.0% (5/50) in 2004 to 16.4% (44/269) in 2010 (p = 0.03), while there was no temporal change for the proportion of methicillin-resistant *S. aureus* observed during the study period (p = 0.90).

### Mortality Associated with CAB

Death within 30 days of admission with an episode of CAB was identified in 5,722 patients, giving an average 30-day mortality over the study period of 37.5% (Table 1). There was an increase in the 30-day mortality over age group from 16.3% in children younger than 1 year of age to 41.5% in those older than 80 years old (p<0.001) (Table 3). Death occurred in hospital in only 51.6% (2,954/5,722) of cases, the remainder occurring after hospital discharge associated with a hospital record of refusal of treatment (30.9%), an improvement in condition (12.2%), transfer to other hospitals (4.9%), or no record of outcome at time of discharge (0.4%). Death in CAB patients occurred rapidly, with 3,384 of 5,107 deaths (59.1%) occurring within the first two days of admission, 481 (8.4%) on day 3, and 353 (6.2%) on day 4. There was a decrease in the 30-day mortality over time from 41.2% in 2004 to 36.0% in 2010 (p = 0.006).

**Table 3:** 30-day mortality associated with community-acquired bacteremia (CAB) in northeast Thailand by age group.

Age (year)	<1	1–9	10–19	20–29	30–39	40–49	50–59	60–69	70–79	≥80
30-day Mortality (%)	16.3	18.6	16.6	29.2	36.7	40.4	41.0	41.2	39.0	41.5

**Figure 3:**
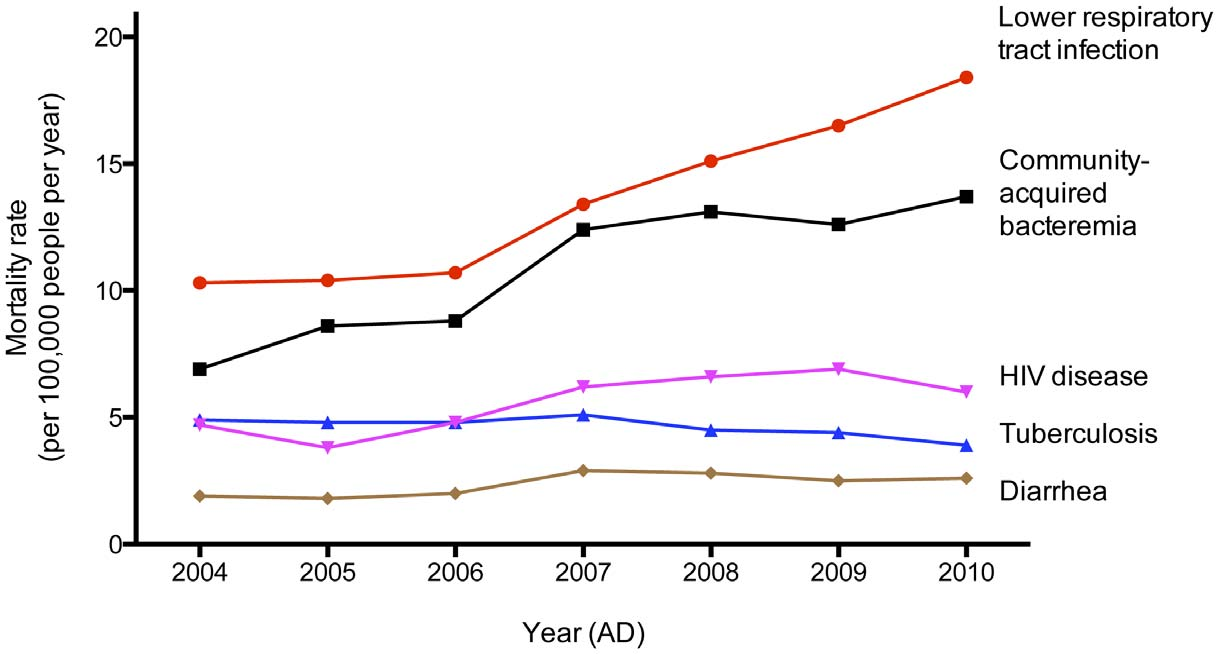
Mortality rates from leading causes of death due to infectious diseases per 100,000 people per year in northeast Thailand between 2004 and 2010. Mortality rate attributable to CAB was calculated as the number of CAB patients who died within 30 days of the admission per 100,000 people per year. Death due to other infectious diseases shown was defined in patients who were admitted to the study hospitals, died within 30 days of admission, and had the primary cause of death based on ICD-10 codes of HIV disease (B20–24), tuberculosis (A15–19), lower respiratory tract infection (J09–18), and diarrhea (A09), after excluding those who died within 30 days due to CAB as described above.

The average mortality rate associated with CAB for northeast Thailand was 11.7 per 100,000 people per year, rising over time from 6.9 in 2004 to 13.7 per 100,000 people in 2010 (p<0.001). Figure 3 shows a comparison between these data and mortality rate associated with other important infectious diseases determined by ICD-10 codes. The rising trend in mortality rate was also observed for lower respiratory tract infection (p<0.001) and HIV disease (p = 0.006), but not for diarrhea (p = 0.41). A decreasing trend in mortality rate was observed for tuberculosis (p<0.001). Death due to malaria and measles were rare during the study period (36 and 1 patients, respectively). In 2010, the mortality rate associated with CAB (13.7 per 100,000 people) was lower than for respiratory tract infection (18.4 per 100,000 people), but higher than other infectious diseases including HIV disease (6.0 per 100,000 people), tuberculosis (3.9 per 100,000 people) and diarrhea (2.6 per 100,000 people).

## Discussion

Our data demonstrate that CAB is an important cause of death in northeast Thailand. Compared to the most common causes of death from infectious diseases, the mortality rate associated with CAB is lower than from respiratory tract infection but higher than other infectious diseases that are important in relation to global heath, including HIV disease, tuberculosis, diarrhea, malaria and measles [17]. Our findings are consistent with data from the United States and Canada, where septicemia is the second most common cause of death from infectious diseases, after lower respiratory tract infection [6], [18]. The number of cases and deaths from CAB presented here are likely to represent a minimum estimate, because we did not account for patients presenting to community or private hospitals that were not referred to the provincial hospitals participating in our study. There are several reasons for the lack of referral, including patients with mild disease and those who were seriously ill and died shortly after admission to district hospitals or during inter-hospital transfer. It is also possible that the diagnosis of CAB could be missed at the provincial hospitals after referral.

An incidence rate for CAB in northeast Thailand of 38.2 per 100,000 people per year in 2010 is much lower than that reported in high-income countries, including 77 per 100,000 people per year in North Jutland County, Denmark, between 1992–1997 [1], 92 per 100,000 people per year in northern Denmark in 2004 [2], and 83 per 100,000 people per year in Olmsted County, USA between 2003 and 2005 [3]. There are very few published studies estimating the incidence rate of community-acquired bacteremia in low and middle-income countries. These include a reported incidence rate for CAB of 4.5 per 100,000 people per year reported in Vientiane, Laos, between 2000–2004 [19], which is very much lower than that observed in our study. By contrast, the incidence of CAB reported for children in Africa is extremely high, and includes 1,457 per 100,000 children per year in children <1 year old in Kilifi, Kenya between 1998–2002 [20], 1,730 per 100,000 children per year in children <1 year old in Manhica District, Mozambique, between 2001–2006 [21], and 1,009 per 100,000 children per year in children aged between 2–29 months in Gambia, between 2000–2004 [22]. This compared with an incidence rate for CAB in children <1 year old of 83.5 per 100,000 people per year in our setting. The 30-day mortality associated with CAB of 37.5% in our setting is much higher than 30-day mortality of 13.0 to 19.0% reported in high-income countries [1], [2], [3], [4], [5], and the in-hospital mortality of 11.0% in Laos [19]. The 30-day mortality for children <1 year old with CAB was 16.3% in our setting, which was comparable to the in-hospital mortality of 10.7% to 28.2% reported in low-income countries in Africa [19], [20], [21], [22]. We found that death attributed to CAB occurred after hospital discharge in 48.2% of fatal cases, most of whom were discharged with a hospital record of refusal of treatment. This reflects a preference amongst people in the study area to die at home. We propose that strengthening of microbiology laboratory infrastructure and national surveillance systems in developing countries is likely to improve the diagnosis of CAB and highlight the importance of CAB as an important cause of death in the future.

To our knowledge, this is the first report of a rising trend of CAB in low and middle-income countries. There was a 129% increase in the incidence rate of CAB between 2004 and 2010 in northeast Thailand, which is much higher than those observed in high-income countries, including a 68% increase in the incidence rate of CAB during 1992–2006 in northern Denmark [2], a 40% increase in the incidence rate of overall bacteremia during 1995–2002 in Finland [23], and 61% increase in the incidence rate of overall bacteremia during 1990–1998 in England and Wales [24]. Our dramatic increase could be due to a combination of an increase in incidence of CAB due to an aging population, an increase in healthcare access as shown by an increase in the admission rate, and an increase in detection of CAB due to improved healthcare practice over time.

During the study period, *E. coli* was the most common pathogen, followed by *B. pseudomallei* and *S. aureus*. The predominance of *E. coli* and *S. aureus* is quite similar to that observed in both high and low income countries [1], [2], [3], [4], [5], [7], [8]. However, *Salmonella enterica* serovar Typhi, the most common cause of CAB in low-income countries [7], [8], is rarely observed in our setting. This could be due to a combination of the nationwide typhoid immunization program which began in 1977 [25], and continuous improvement in sanitation in Thailand [26]. *B. pseudomallei*, the cause of melioidosis, is also a common cause of CAB in Australia [27], and an important cause of CAB in Laos [19]. This Gram-negative bacillus is present in the environment in many tropical countries and infection is most often reported from South and East Asia and Northern Australia [16]. Melioidosis is predicted to be under-diagnosed in many developing tropical countries because of a combination of lack of diagnostic clinical features, microbiology facilities and expertise in bacterial identification [16]. As *B. pseudomallei* is intrinsically resistant to commonly used first line antimicrobials in tropical settings for sepsis including penicillin, aminoglycosides and most cephalosporins [28], empirical regimens involving ceftazidime or a carbapenem drug are required if melioidosis is suspected to be a cause of CAB in northeast Thailand [16]. Prevention of melioidosis includes avoidance of direct contact with soil and standing water and washing after exposure [16], while prevention of *E. coli* bacteremia may need to focus on prevention of community-acquired urinary tract infection [29], [30]. The low incidence of CAB due to MRSA in Thailand is consistent with our previous report [12]. However, the rising trend of ESBL-producing *E. coli* and *K. pneumoniae* as a cause of CAB is of concern [31], [32], [33]. Current usage of antimicrobials in Thailand, and the spread of ESBL-producing *Enterobacteriaceae* in animals and the environment are worthy of investigation.

The major limitation of this study is that complete clinical data was not available for further analysis. Another potential limitation is that blood culture may not have been performed for all patients with a likelihood of bacteremia, and this might lead to a difference in the incidence of CAB among participating hospitals. We propose that strengthening of guidelines for the diagnosis of CAB and an increased use of blood culture is likely to disclose underestimations of CAB in some areas [10], [11].

Although monitoring of infectious diseases in developing countries is hampered by incomplete routine notifications and lack of advanced research facilities, our study shows that careful evaluation of readily available routine databases can provide useful information on the rate of serious bacterial infections. The methodology used in our study could be applied to other geographical areas where microbiological facilities are available to provide a more comprehensive global picture of the importance of CAB as a cause of death.
