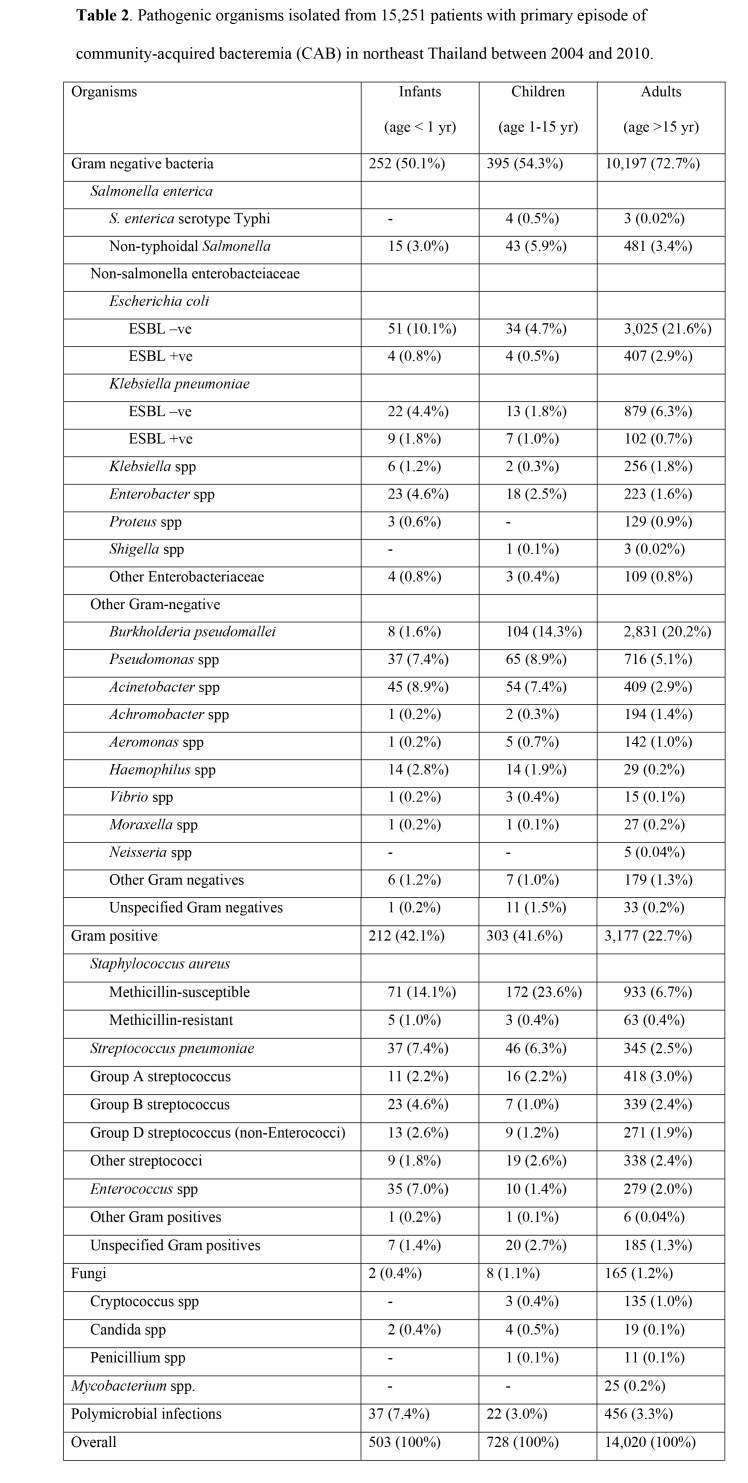# Correction: Epidemiology, Microbiology and Mortality Associated with Community-Acquired Bacteremia in Northeast Thailand: A Multicenter Surveillance Study

**DOI:** 10.1371/annotation/e199ebcc-0bc1-4be1-ad91-ad2a8c0c9382

**Published:** 2013-10-16

**Authors:** Manas Kanoksil, Anchalee Jatapai, Sharon J. Peacock, Direk Limmathurotsakul

An extra column was erroneously added to Table 2. Please see the correct Table 2 here: 

**Figure:**